# Nature- based nursing intervention: the impact on insomnia and feeling of hopelessness among patients with depression at Nour El-Hikma psychiatric hospital, Egypt (A controlled quasi- experimental study)

**DOI:** 10.1186/s12888-026-07860-1

**Published:** 2026-02-21

**Authors:** Hanaa A. Radwan, Nehal Sobhy Emaraa, Rania Sobhy El gendy, Kariema I. El Berry

**Affiliations:** Department of Psychiatric Mental Health Nursing, Faculty of Nursing, Menoufia University, Shibin El Kom, 32511 Egypt

**Keywords:** Nature-based nursing intervention, Insomnia, Feeling of hopelessness, Patients with depression

## Abstract

**Background:**

Depression is the most prevalent mental disorder in Egypt. It is often accompanied by insomnia and feeling of hopelessness which significantly cause many challenges. Traditional psychotherapy does not alleviate these symptoms. Nature- based nursing intervention, also referred to as a green intervention, that is implemented as program in a natural environment. It has emerged as favorable intervention to enhance mental health outcomes. Conversely, their impact on improving insomnia and hopelessness remain unclear.

**Aim:**

The study evaluated how does a nature-based nursing intervention affects insomnia and feelings of hopelessness in patients with depression.

**Methods:**

A quasi-experimental research design was handled from half of May 2025 to half of October 2025 on 62 depressed patients classified into study and control groups at the Nour El-Hikma psychiatric hospital, Meet-Khalaf, Shibin Elkom, Menoufia Governorate, Egypt. Insomnia and feeling of hopelessness were measured utilizing a sleep disorders scale, and a Beck Hopelessness Scale, respectively. The SPSS v26 software was chosen to perform the data analysis. Categorical data were expressed as frequencies and percentages, and the Chi-square test was applied to find out group differences.

**Results:**

There was no significant difference between the two groups in terms of sociodemographic variables (*P* < 0.05). There was a statistically significant difference between depressed patients in the studied groups prior, post, and follow-up after 2 months regarding insomnia level and feeling of hopelessness (*p* < 0.001). Also, there was a statistically significant positive correlation between hopelessness and insomnia after nature-based nursing intervention among only the nature-based group (*r* = 0.977*, *p* = 0.005).

**Conclusion:**

Nature-based nursing intervention had significant benefits on improving insomnia and reducing feelings of hopelessness in patients with depression.

**Clinical trial:**

No clinical trial.

**Supplementary Information:**

The online version contains supplementary material available at 10.1186/s12888-026-07860-1.

## Introduction

Waking up burdened with hopelessness, unable to sleep at night or face the day, is a live reality for millions of people with depression across the globe [1]. Depression is more than mere instability in mood or transitory emotions. It represents an internal dispute that results in senses of downcast, sad mood, inadequate sleep, guilt, and emptiness and in severe cases, hopelessness and suicidal ideation. Approximately 280 million individuals globally contend with this challenge. By 2030, it is predicted to become one of the primary determinants of health disability globally [2].

The key symptom of this disorder is the sleep disturbance; maybe that is why the depressed patients first seek help. It encompasses insomnia, narcolepsy, sleep- disordered breathing and restless legs syndrome [3]. In patients with depression, insomnia is the most common sleep disturbance, impacting 80% to 90% of individuals [4]. It presents as challenges in initiating or maintaining sleep, excessive daytime drowsiness, nocturnal awakenings [5], difficulties with concentration, and reduced energy levels. The symptom is also a major predictor of depressive relapses and may lead to increases in the risk of suicidal behavior and hopelessness, which results in unfavorable clinical outcomes [3].

Depression is not limited to sleep disturbances but also manifested in feelings of hopelessness, which are among its most prominent and dangerous features [1]. Hopelessness is not just a passing feeling of pessimism. Rather, it is a profound cognitive and emotional state that robs an individual of their sense of ability to change and makes them see the future as bleak and devoid of opportunities [6]. Its danger lies in its potential as a gateway to depression and suicidal behavior, as emphasized by the hopelessness theory. In addition, it serves as a significant indicator of a poor response to treatment [7]. Wherefore, early intervention to handle insomnia and feelings of hopelessness is vital for protecting mental health and enhancing recovery opportunities.

In this context, nature-based nursing intervention (NBNI) emerges as one of the most promising emerging trends in mental health. NBNI is the term used to describe planned intervention and activities that involve direct sensory and physical interaction with natural elements in parks, forests, or gardens [8]. The NBI theory was supported by Wilson’s bipohilia hypothesis [9], which claims that human beings possess an instinctive connection to nature, as well as attention restoration theory [10], which suggests that spending time in natural environments minimizes rumination and recovers cognitive resources. Furthermore, stress reduction theory [11] pointed out that the effect-regulating potential of natural environments leads to promoting relaxation and reducing physiological arousal.

NBNIs include activities like walking in nature, gardening, exposure to natural light, listening to nature sounds, or receiving psychological support in natural settings [12]. Being involved with nature produces relaxation and a sense of belonging to the environment and oneself while reducing negative emotions like hopelessness, according to the result of Van den Berg & Beute [13]. A natural place can stimulate the secretion of melatonin and endorphin production, which results in deep, restful sleep and high feelings of pleasure and empowerment, as well as improve memory, cognition, and concentration [14–17]. Furthermore, the natural environment can help hopeless people to promote positive thinking by reframing their negative thoughts through using the growth of plants as metaphors and hope boxes for hope and continuity [18, 19].

Psychiatric nurses are crucial in the formulation, implementation, and valuation of nature-based interventions for insomnia and hopelessness as a way to improve patients’ mental health outcomes [20]. Their responsibilities incorporate thorough evaluation, education, support, ongoing direction, and leadership [21]. In addition, they work as a member of a variety of teams that act as champions for the adoption of cost-effective nonpharmacological interventions into mental healthcare.

## Significance of the study

According to surveys, the prevalence of depressive illnesses has increased from almost a quarter to three-quarters of the population, making them the most common mental disorders in Egypt [22, 23]. Depression symptoms like insomnia and hopelessness constitute a major health and economic challenge, as they are associated with substance abuse as well as limited employment and educational prospects [24]. In addition to poor response to treatment, aggressive behavior increases pressure on families and society [25]. Furthermore, the economic strain of major depression in Egypt is estimated at 7 billion Egyptian pounds due to the price of medications, hospitalization, and costly treatments [26]. Accordingly, the requirement for more impactful and innovative intervention choices is highlighted.

Despite the growing evidence of the importance of natural environments in enhancing mental well-being, studies that have investigated in-depth their influence on insomnia and hopelessness in patients with depression persist restricted, representing a research gap that merits being examined. Handling this gap is a crucial initial step in raising the standard of care because, in addition to their ability to reduce insomnia and boost optimism, nature-based interventions are also relatively reasonably priced considering Egyptian culture. Moreover, we expect the results of this study to benefit multiple sectors. They will give clinical practitioners choices for supportive interventions that are easy to incorporate into care plans and directly help patients and their families by reducing the psychological and financial costs associated with depression. Further, it will assist healthcare decision-makers in establishing more careful and competent strategies. Therefore, this study aimed at exploring the effectiveness of a nature-based nursing intervention to improve insomnia and alleviate feelings of hopelessness in patients with depression.

## Subjects and methods

### The purpose of the study

Appraise the effectiveness of a nature- based nursing intervention on insomnia and feeling of hopelessness among patients with depression at Nour El-Hikma Psychiatric Hospital, Egypt.

### Hypothesis

#### H1

The patients who engage in nature- based nursing intervention will experience lower hopelessness and lower insomnia scores after nature- based nursing intervention than patients who don’t engage in nature- based nursing intervention.

#### Research design

A quasi-experimental design utilizing pre, posttests and follow-up (a study and control group) to appraise the effectiveness of nature-based nursing intervention. The research aimed at improving feelings of hopelessness and insomnia among depressed patients through ten structured intervention sessions.

### Research setting

The Menoufia governorate consists of 9 main centers. The current study was performed in the Shibin-Elkom center, Menoufia governorate. Shibin-Elkom center was selected randomly from a container containing all names of Menoufia centers. Shibin-Elkom center consists of 8 villages. Meet-Khalf (Austobary) village was selected randomly from a container containing all names of Shibin-Elkom villages. The resulting selection was Nour El-hikma psychiatric hospital. The primary setting as Nour El-hikma psychiatric hospital was selected for data collection, as it offers the right environment and appropriate resources to support the current research.

#### Subjects

A purposive sample of 62 depressed patients selected from Nour El-Hikma psychiatric hospital, Menoufia governorate, Egypt, from half of May to half of October (2025). All eligible patients who fulfilled the inclusion requirements during the data collecting period were men. As a result, all the patients in the study sample were men.

### Inclusion criteria included

Subjects had a diagnosis of depressive illness (as determined by the initial medical diagnosis on admission to the hospital and agreed to participate in the study. Furthermore, subjects had feelings of hopelessness and insomnia. These symptoms were confirmed using validated instruments such as the Beck Hopelessness Scale for hopelessness and the Sleep Disorders Scale for insomnia. Subjects had to be within the adult age range of 18 to 65 years old. This demographic was selected given that most individuals within this age span are susceptible to and suffer from major depressive disorder [27]. In addition, subjects with chronic physical illnesses such as hypertension and diabetes were included, as these conditions don’t limit their ability to engage in intervention activities.

### Exclusion criteria

Patients with active suicidal ideation were typically excluded due to the high clinical risk they present and the ethical obligation to ensure their safety, or they require safety plans. In addition, patients with cognitive impairment were excluded. This exclusion was made to ensure participants could comprehend and participate with the study’s assessment and intervention, as cognitive impairment may influence this capacity and skew the results. Additionally, patients with physical disability were excluded, as our nature -based nursing intervention needs patient physical ability to perform a natural activity such as planting and playing.

### Depressed patients’ sample size and sampling technique

**Fig. 1:**
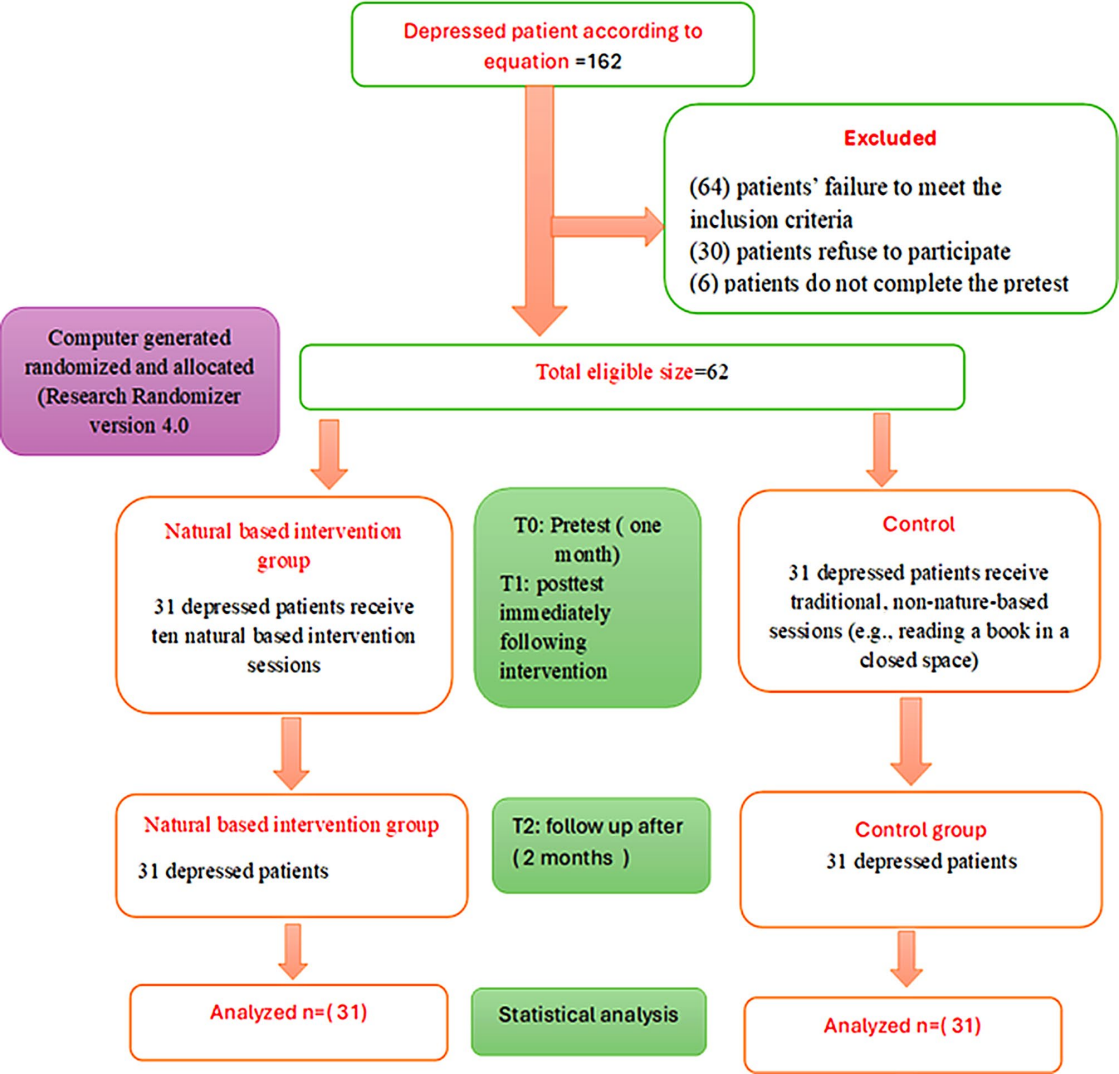
Evaluation of the enrollment and recruitment procedure for patients with depression

Based on the study by Buysse et al. [28], the mean insomnia score among patients with depression was 10.41, with a standard deviation of 3.24. The sample size was calculated using the following equation: Z₁-α/22SD2/d² [29], where Z₁-α/2 is the standard normal variate, SD is the standard deviation of the variable under study, and d is the absolute error or precision. Assuming a confidence level of 95% (Z = 1.96) and a margin of error of 1. Therefore, the minimum required sample size was approximately 162 participants. During the sample screening phase, a large number of patients were evaluated to verify their compliance with the study’s inclusion criteria. (64) patients were excluded due to their failure to meet the inclusion criteria. In addition, 30 patients refused to participate in the study after its objectives and procedures were explained, for personal reasons or due to unwillingness to commit to the sessions. Also 6 patients do not complete the pretest. Thus, the study was completed with the final sample of 62 patients who met the criteria and agreed to voluntarily participate. Written informed consent was obtained from all participants before randomization. Participants were randomly assigned into two groups (a nature-based group and a control group) utilizing a program called “Research Randomizer version 4.0.” A nature based group: Includes (31) patients who received nature-based nursing intervention. Control group: Includes (31) who undergo traditional, non-nature-based sessions (e.g., reading a book in a closed space) as illustrated in (Fig. 1). The investigator visited Nour El-Hikma psychiatric hospital two days a week (Wednesday and Thursday) for 5 months (assessment and implementation phase) (from half of May 2025 to half of August 2025) until the investigator reached the required size and two months for follow-up (from half of August 2025 to half of October 2025).

### Avoid contamination between groups (the control group and a natural based group)

The researcher coordinated with the hospital management team to prevent overlaps between the two groups by:

- A request for formal support was obtained from the hospital administration and nursing team to organize a separate schedule for each group (park time, mealtime, and gathering time).
- Assign a supervisor to each group to help monitor individuals and prevent them from mingling, even during informal times.
- Create a separate schedule for each group so that each group has its own time for activities, homework, and recreation.
- Prepare sample awareness text for research participants: It stipulates not to discuss the various activities and treatment methods that any group is going through with members of the other group and to adhere to the time frame specified for each activity for each group separately.

### Measure instruments

#### Structure interviewing questionnaire

After a widespread literature review (Zein-elabdeen, Ibrahim, & El-Bilsha [30]; Dafallah [31], a sociodemographic and medical history assessment questionnaire was created. Demographic assessment involved sex, age, residence, educational level, and marital status. The medical history assessment included family history, history of suicide ideation, illness history, and physical disease.

#### Beck hopelessness scale (BHS)

It was originally developed by Beck, Weissman, Lester, & Trexler [32] to evaluate one’s negative expectations about the future. We used a validated Arabic scale translated by Michael [33] which had a reliability coefficient of 0.78, and the internal consistency (Cronbach coefficient alpha) of the (BHS) was 0.90, while in the current study it was 0.85. This scale involved only 20 statements, each of which has only two possible answers: yes or no. The probability of yes is given a score of 1, and the probability of no is given a score of 0, considering that there are nine items that are scored in the opposite direction, which are (1,3,5,6,8,10,13,15,19). The scores range from 0 to 20. Higher BHS ratings indicate more pessimistic outlooks for the future. A score of ten or higher denotes extreme despair and a higher risk of suicide. The total scoring system of BHS represented as the following:0–3 = mild hopelessness, 4–8 = moderate hopelessness, 9–14 = severe hopelessness, and 15–20 extremely severe hopelessness.

#### Sleep disorders scale (insomnia)

The Arabic version of the scale was originally developed by Abdul Halim [34]. It consists of 24 items designed to assess four dimensions of sleep disorders: insomnia, nightmares, night terrors, and narcolepsy. In the current study we used the insomnia dimension to measure the severity of insomnia symptoms among participants. This dimension consists of 6 items (1,5,9,13,17,21) rated on a 3 - point Likert scale ranging from 1to 3: never (1), sometimes (2), and always (3). The total score for this dimension ranges between 6 to18. Accordingly, insomnia was categorized as follows: mild (6 to < 10 points), moderate (10 to < 14 points) and severe (≥ 14 points). The internal consistency of the insomnia dimension was good (Cronbach’s alpha = 0.75) [34]. This dimension was specifically chosen because it is directly related to the symptoms targeted in this study, and its use without the other dimensions helps reduce the burden on participants without affecting the reliability of the results related to insomnia. In the current study the Cronbach’s alpha of the insomnia dimension is 0.85.

### Procedure

The research began in the first half of May and finished in the first half of October (2025). The research’s intervention took place in four different phases:

#### Pre-assessment phase

To formulate the intervention, the researcher conducted a thorough review of nature intervention and ecotherapy literature [35, 36] to identify effective activities that psychological studies have proven to have a positive impact on improving mental health, especially in reducing symptoms of depression. The researchers conducted an informal focus group discussion with a select group of patients to understand their attitudes regarding the intervention and the nature-based activities they preferred. Most of them expressed a strong willingness to participate in the intervention. Matched with the intervention’s goals of reducing insomnia and feeling of hopelessness as well as soothing properties and ease of care, an agricultural engineer was consulted to choose proper plants, such as jasmine and basil. There was document evidence that jasmine is recommended because its natural scent can enhance relaxation at the mitigation of sleep [37]. Moreover, basil (Ocimum basilicum) is known to improve insomnia and enhance mood, hope, and psychological well-being due to its neuroprotective and anxiolytic properties [38, 39]. Recycled planters and other lightweight, safe materials were used to supply the necessary equipment.

To avoid unstable weather conditions, the intervention was implemented during the summer months (May–August). Official agreements were acquired from the Faculty of Nursing at Menoufia University and the hospital administration. A pilot study was run on 10% of the sample to investigate the legibility of the tools and the applicability of the intervention, and feedback contributed to its improvement.

#### Assessment phase

Eligible patients were selected after the review of their medical records, and after obtaining written informed consent, in-person interviews were performed. Based on the patient’s ability to talk and understand, the first interview was 40 to 60 min, and three assessment tools were administered.

#### Implementation phase

Study participants received an introduction to the study itself. After this, the researcher explained the objectives of the study and answered questions. Thereafter, additional demographic information was completed, after which baseline measurements were administered. The participants in the intervention group were further divided into six subgroups (G1-G6) with 10–11 patients in each group. The investigator implemented sessions initially for subgroups G1, G2, and G3 on Wednesday, while on Thursday, the investigator implemented sessions for subgroups G4, G5, and G6. The group intervention program was conducted for a total duration of 10 weeks, with 1 session each week per group lasting 60 min per session. To create a supportive natural environment, sessions were held in the garden of the hospital while maintaining safety precautions as well as environmental consistency. There was a total of ten sessions: including one introductory session, eight nature-based activities intervention sessions, and one final evaluation session. Objectives of the sessions were to facilitate active participation with nature in psychosocial exercises to alleviate the participant’s symptoms of insomnia and feeling of hopelessness. The sessions included a set of regular and planned activities, including:

**1st session: Preparation and building the therapeutic relationship**

Introducing participants to the program’s purpose and psychological benefits, and creating a supportive and safe environment, the session aimed to reduce anticipatory anxiety and enhance a sense of belonging.

**2nd session: Exercises in nature**

The session emphasizes light exercises, such as walking and jumping rope in the park, squat jumps, and light jogging to improve sleep quality through improving mood and increasing serotonin secretion, which supports relaxation.

**3rd and 4th session: Gardening**

Patients participated in agricultural farming and planting jasmine and basil plants, using agriculture as a symbolic tool to reconstruct negative thoughts into more positive ones.

**5th session: Nature meditation (part one)**

The session aimed at reducing excessive thinking and improving sleep quality through encouraging patients to use relaxation techniques and connect with the present moment, such as lying on the grass and mindful walking.

**6th and 7th sessions: Natural meditation (Part two)**

To cultivate gratitude and progressively restore hope, the sessions focus on mental exercises, including the hope box, positive memory meditation, and the “Three Good Things” exercise.

**8th session: Nature-based play (Part 1)**

This session comprised games like sketching and tug-of-war, played both individually and in groups, were utilized as nonverbal means of expressing feelings, converting psychological suffering into tangible accomplishments, and fostering hope.

**9th session: Nature-based play (Part 2)**

To reduce feelings of hopelessness associated with depression, this session focused on interactive games such as football games and bubble blowing that increase self-confidence and a sense of accomplishment.

#### Evaluation phase

**10th session: closure of the intervention and evaluation of the effectiveness of a nature- based nursing intervention**

The researcher conducted a comprehensive review of all activities carried out during the intervention sessions. The researcher identified the benefits that occurred in the participants’ condition regarding insomnia and feelings of hopelessness immediately after completing the intervention and after 2 months (follow-up) by applying the assessment instruments (BHS, Sleep Disorders Scale (Insomnia subscale) for the intervention group and control group.

### Nature based intervention follow up

During the two-month follow-up period, the researchers maintained regular communication with the nursing team, who had been part of the study from the beginning and were responsible for the patients’ care. In addition, they visited the participants twice (one visit each month).

These visits aimed to monitor participants’ adherence, reinforce the techniques learned, and provide clarification or guidance when participants encountered difficulties in practicing the sessions (e.g., misunderstanding a meditation step or reduced motivation). To ensure that any improvements noticed could be attributed solely to the implemented nature-based intervention, the researchers coordinated with the treating physicians to ensure that no changes were made to the type or dosage of medications throughout the intervention and follow-up periods. Also, to minimize contamination between nature-based intervention and control groups, measures were taken. Participants consistently conducted nature-based intervention at various times and were assigned to different wards. All participants were also instructed not to share details of their interventions with patients in the other group.

### Ethical issues and approval

This study complies with the Declaration of Helsinki guidelines for approval of an ethics committee opinion. Ethical approval for this research was gained from the Ethical and Research Committee of the Faculty of Nursing, Menoufia University (approval number: 1000/7/5/108/25). Official permission was attained from the hospital director to run the study after clarifying the purpose of the study. Before participants were involved in the study, they were informed about its purpose, methods, potential risks, and benefits and their informed consent was acquired. Anonymity and confidentiality of the subjects’ data (use code for patient name, all data stored in a safe location and only shared with authorized personnel). Also, the participants were informed that their involvement in this study is completely optional; they have the right to choose to participate in the study and cancel their participation at any point in time.

### Statistical analysis

Statistical analysis was managed using SPSS 26.0. Kolmogorov-Smirnova and Shapiro-Wilk tests were used for normally distributed data; however, Mann-Whitney and Kruskal-Wallis H tests were utilized for non-normally distributed data. Mean (X) and standard deviation (SD) were used to represent quantitative data, while the qualitative data was presented in the form of frequency distribution tables, numbers, and percentages. To compare categorical data between the intervention and control groups, the Chi-square test (χ²) was applied. To value the relationship between continuous or ordinal variables, the Spearmen correlation coefficient test (r) was utilized. The level of significance was set as a P value ≤ 0.05 for all significant tests.

## Results

Table 1 summarizes baseline sociodemographic variables of depressed patients in the group studied and all of whom were male. The largest proportion of studied patients from both groups was in the age cohort of > 44 years, with 58.1% of patients in the nature- based group and 64.5% of patients in the control group. Regarding education, secondary education constituted the highest proportion, with 61.3% in the nature- based group and 58.1% in the control group. Regarding living situation, a smaller percentage of patients in the control group lived alone 29%, while the majority of them were in a nature- based group and a control group respectively living with their family 67.7%,77.4%. As regards occupational status, unemployment constituted the highest proportion, with 87.1% in the nature- based group and 77.4% in the control group. In terms of marital status nearly half of the patients studied in both groups were single. Generally, sociodemographic characteristics did not show any statistically significant differences between the two groups (*P* > 0.05).

Table 2 describes the baseline medical history of depressed patients in the studied group. The table simplified that the largest percentage of patients had more than one psychiatric hospital admission (64.5% in the control group versus 74.2% in the nature- based group), while a smaller percentage were first-time admissions. The largest proportion of patients studied (93.5% in the nature- based group and 80.6% in the control group) had a positive family history of illness. None of patients studied in both groups (nature-based and control groups) reported any chronic physical disability and suicidal ideation. Overall, baseline medical history did not demonstrate any statistically significant differences between nature and control groups (*P* > 0.05).

Table 3 displays insomnia levels of depressed patients in the studied groups. Initially, the two groups were similar in the distribution of insomnia levels (mild, moderate, severe) at baseline, with no statistically significant differences. At post-intervention, significant enhancement was apparent in the nature- based group, represented by 51.6% of the nature- based group having mild insomnia, and “no patients reported severe insomnia compared to minimal improvement (16.1%, 45.2%) of the control group with clear statistical significance (P = 0.003). After a 2-month follow-up, 32.3% of the patients in the nature- based group reported mild insomnia, while 12.9% of patients in the nature-based group relapsed to severe insomnia (p = 0.000*). Contrariwise, the control group reported slight change over time, with the highest proportion evident in severe or moderate categories. These outcomes illustrate that nature- based nursing intervention has benefits in the short term, suggesting the need to add additional support strategies to maintain long-term results.

Table 4 illustrates the levels of hopelessness across the nature- based group and control groups at pre-post and 2 months following nature- based nursing intervention. There was no evident variation between the two groups in terms of severe and extremely severe hopelessness prior to the intervention (*p* = 0.437). Following the intervention, more than half of patients in the control group remained at the severe level (58.1%), and more than one third experienced extremely severe (38.7%) levels of hopelessness. Dissimilarly, the nature- based groups who complained of extremely severe hopelessness diminished from 35.5% to zero, and most patients progressed to the moderate level (77.4) (p value = 0.000*). During the two-month follow-up, a tenuous increase in severe hopelessness levels was seen in the nature- based group; however, the majority of participants stayed at the moderate level, with no patients classified as extremely severe. Importantly, these observations were still markedly better compared with baseline and with the control group. This pattern suggests that while nature-based nursing intervention produces substantial short-term benefits, reinforcement strategies may be required to sustain improvements in the longer term.

**Fig. 2:**
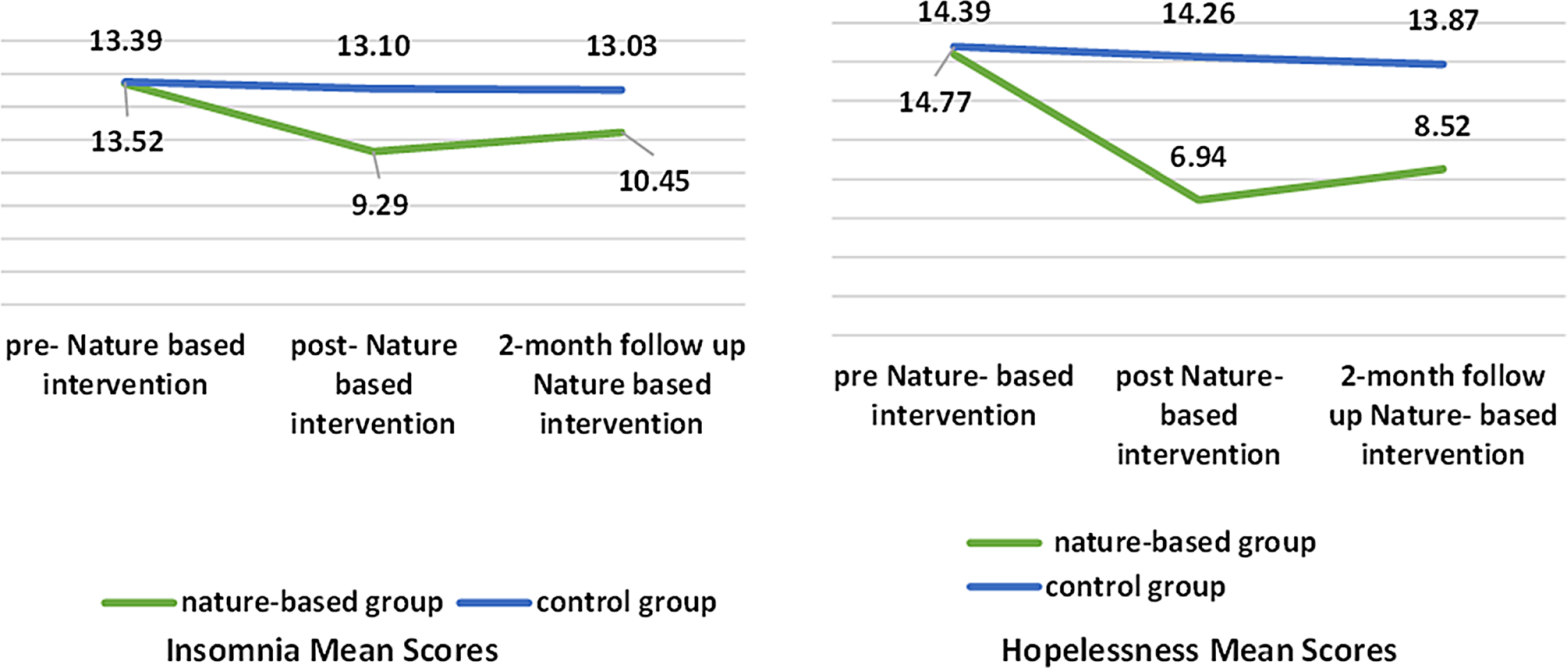
Comparative mean scores of insomnia and hopelessness of depressed patients in the studied groups (*n* = 62)

Figure 2 demonstrates the mean scores of insomnia and hopelessness across the nature- based and control groups at pre-post and 2 months following the nature- based nursing intervention. Pre nature- based nursing intervention, both groups mean scores of insomnia and hopelessness did not differ significantly (*p* = 0.949 and 0.439, respectively). In dissimilarity, the nature- based group’s mean insomnia score came down from 13.39 ± 3.61 to 9.29 ± 1.97 post-intervention with a minor increase at two-month follow-up to 10.45 ± 2.03, while the control group displayed persistently high scores. In a similar manner, the nature- based group’s hopelessness means scores fell from 14.77 ± 1.96 to 6.94 ± 1.82 after the intervention with minimal increase at the two months follow up to 8.52 ± 2.11, but the control group’s scores were elevated during the study period. This finding reflects a substantial decrease in the study group’s mean insomnia and hopelessness scores compared to the control group, confirming the short-term effectiveness of the applied intervention.

Table 5 demonstrates the correlation between feeling of hopelessness and insomnia among the nature-based group after and 2 months follow up to a nature- based nursing intervention. Feeling of hopelessness at post-intervention was positively correlated with insomnia post intervention (*r* = 0.977*, *p* = 0.005). Moreover, this positive correlation persisted between feeling of hopelessness at post-intervention and insomnia at the two-months follow-up (*r* = 0.958*, *p* = 0.010).

Table 6 reflects associations between insomnia and feeling of hopelessness with socio-demographic variables in the nature- based and control groups (*n* = 62). This table shows a substantial relation between insomnia and living arrangements (with family or alone; *p* < 0.001), education level (K = 16.796, *p* = 0.000*), and marital status (K = 17.031, *p* < 0.001).Significant relations were also seen between feeling of hopelessness and age (K = 14.903, *p* = 0.001), marital status (K = 7.986, *p* = 0.018), occupation (U = 164, *p* = 0.025), and illness history (U = 197.5, *p* = 0.001). On the other hand, no noteworthy association was found between either group’s residency, family history of depression, or history of suicide.

**Table 1:** Baseline sociodemographic variables of depressed patients in the studied group (*N* = 62)

Studied variables		Nature- based group (*n* = 31)		Control group (*n* = 31)		X^2^	*P* value
		*N*	(%)	*N*	(%)		
Age	≤ 28.00	2	(6.5)	2	(6.5)	0.305	.858^ns^
	29.00–43.00	11	(35.5)	9	(29.0)		
	≥ 44.00	18	(58.1)	20	(64.5)		
	X̄ ±SD	43.48 ± 7.47		44.16 ± 8.58			
Education	Basic education	6	(19.4)	6	(19.4)	0.104	0.949 ^ns^
	Secondary education	19	(61.3)	18	(58.1)		
	University education	6	(19.4)	7	(22.6)		
Marital status	Single	14	(45.2)	13	(41.9)	1.762	0.414 ^ns^
	Married	4	(12.9)	8	(25.8)		
	Divorced	13	(41.9)	10	(32.3)		
	Widowed	0	(0.0)	0	(0.0)		
Occupation	Employment	4	(12.9)	7	(22.6)	0.995	0.319 ^ns^
	Un employment	27	(87.1)	24	(77.4)		
Residence	Urban	11	(35.5)	16	(51.6)	1.640	0.200 ^ns^
	Rural	20	(64.5)	15	(48.4)		
**Living Situation**							
Do you live with your family?	Yes	21	(67.7)	24	(77.4)	0.729	0.393 ^ns^
	No	10	(32.3)	7	(22.6)		
Do you live alone?	Yes	0	(0)	9	(29.0)	-	-
	No	31	(100)	31	(100)		

X2: chi−square test; ns: not significant; − No Statistic could be computed because one of the cells has zero value

Note: All subjects were male; therefore, sex was not included as a variable

**Table 2:** Baseline medical history of depressed patients in the studied group (*N* = 62)

Studied variable		Nature- based group (*n* = 31)		Control group (*n* = 31)		X^2^	*P* value
		*N*	(%)	*N*	(%)		
Patient Admission History	First admission to a psychiatric hospital	8	(25.8)	11	(35.5)	0.683	0.409 ns
	More than one admission to a psychiatric hospital	23	(74.2)	20	(64.5)		
Do you have any chronic physical disability?	Yes	0	(0)	0	(0)	0.081	1.00 ns
	No	31	(100)	31	(100)		
“Is anyone from your family suffering from depression?”	Yes	29	(93.5)	25	(80.6)	2.296	0.130 ns
	No	2	(6.5)	6	(19.4)		
**History of suicide ideation**							
Have you ever experienced thoughts of suicide?	Yes	0	0	0	0	0.00	1.00 ns
	No	31	(100)	31	(100)		

ns: non-significant; X^2^: chi-square test

**Table 3:** Insomnia levels of depressed patients in the studied groups (n = 62)

Insomnia Levels	Pre- Nature- based intervention						Post- Nature-based intervention						2-month follow up nature- based intervention					
	Nature based group (n=31)		Control group (n=31)		X^2^	*P* value	Nature based group (n=31)		Control group (n=31)		X^2^	*P* value	Nature based group (n=31)		Control group (n=31)		X^2^	*P* value
	N	(%)	N	(%)			N	(%)	N	(%)			N	(%)	N	(%)		
Mild	4	12.9	4	12.9	.000	1.000	16	51.6	5	16.1	8.713	0.003*	10	32.3	4	12.9	18.303	0.000*
Moderate	10	32.3	10	32.3			15	48.4	12	38.7			17	54.8	11	35.5		
Severe	17	54.8	17	54.8			0	.0	14	45.2			4	12.9	16	51.6		

X^2^: chi-square test; *: Statistically significant at P≤0.05

**Table 4:** Hopelessness severity levels of depressed patients in the studied groups (n = 62)

Hopelessness levels	Pre- Nature- based intervention						Post- Nature- based intervention						2-month follow up Nature- based intervention					
	Nature based group (n=31)		Control group (n=31)		X^2^	*P* value	Nature based group (n=31)		Control group (n=31)		X^2^	*P* value	Nature based group (n=31)		Control group (n=31)		X2	*P* value
	N	(%)	N	(%)			N	(%)	N	(%)			N	(%)	N	(%)		
Mild	0	0	0	0	.603	0.437	2	6.5	0	.0	42.508	0.000*	1	3.2	0	.0	39.143	0.000*
Moderate	0	0	0	0			24	77.4	1	3.2			22	71.0	0	.0		
Severe	20	64.5	17	54.8			5	16.1	18	58.1			8	25.8	20	64.5		
Extremely sever	11	35.5	14	45.2)			0	.0	12	38.7			0	0	11	35.5		

X^2^: chi-square test; *: Statistically significant at P≤0.05

**Table 5:** Correlation between feeling of hopelessness, insomnia among only nature- based group *n* = 31 (post nature- based intervention and 2 months follow up)

Study Variables	Feeling of Hopelessness (post-intervention)	Feeling of Hopelessness (2-month)
Nature- based group (n = 31)		
Insomnia (post)	*r* = 0.977*, *p* = 0.005	*r* = 0.171, *p* = 0.357
Insomnia (2-month follow-up)	*r* = 0.958*, *p* = 0.010	*r* = 0.162, *p* = 0.385

r: Spearmen correlation coefficient; *: Statistically significant at *P* ≤ 0.05

**Table 6:** Associations of insomnia and feeling of hopelessness with socio-demographic variables among the study groups(*n* = 62)

Sociodemographic characteristics		Insomnia						Feeling of Hopelessness					
		Nature- based group (*n* = 31)		Control group (*n* = 31)		Test of Sig	*P* value	Nature- based group (*n* = 31)		Control group (*n* = 31)		Test of Sig	*P* value
		X̄	SD	X̄	SD			X̄	SD	X̄	SD		
Age	≤ 28.00	16.00	0.00	16.00	0.00	K = 4.778	0.092	9.00	0.00	16.00	0.00	**K = 14.903**	0.001*
	29.00–43.00	12.18	4.40	12.00	4.33			15.10	1.55	12.00	4.33		
	≥ 44.00	13.83	3.11	13.95	2.89			14.89	1.90	13.95	2.89		
Sex	Male	13.39	3.61	13.52	3.39	-	-	14.39	1.96	14.77	2.55	-	-
	Female	-	-	-	-			-	-	-	-		
Education	Basic education	9.67	1.51	10.83	1.83	**K = 16.796**	0.000*	-	-	-	-	K = 4.652	0.098
	Secondary education	13.63	3.61	13.72	3.49			14.19	2.31	13.72	3.49		
	University education	16.33	1.37	15.29	3.04			15.85	1.99	15.29	3.04		
Patient Admission History	First admission	13.50	4.81	13.00	4.24	U = 393	0.811	15.79	1.65	13.00	4.24	**U = 197.5**	0.001*
	More than one admission	13.35	3.23	13.80	2.91			14.05	2.31	13.80	2.91		
Marital status	Single	15.71	2.23	15.23	2.28	**K = 17.031**	0.000*	14.41	2.80	15.23	2.28	**K = 7.986**	0.018*
	Married	11.00	5.77	12.25	4.68			15.92	1.93	12.25	4.68		
	Divorced	11.62	2.69	12.30	2.67			14.09	1.35	12.30	2.67		
Occupation	Work	12.00	4.62	13.29	3.73	U = 249.5	0.565	16.18	2.32	13.29	3.73	**U = 164**	0.025*
	Not work	13.59	3.50	13.58	3.37			14.24	2.12	13.58	3.37		
Residence	Urban	14.45	4.25	13.81	3.83	U = 344	0.066	14.89	2.01	13.81	3.83	U = 431	0.537
	Rural	12.80	3.17	13.20	2.96			14.34	2.45	13.20	2.96		
Do you live with your family?	Yes	14.81	3.44	14.29	3.41	**U = 114**	0.000*	14.76	2.52	14.29	3.41	U = 306.5	0.209
	No	10.40	1.58	10.86	1.57			14.12	1.32	10.86	1.57		
Do you live alone?	Yes	11.17	2.29	11.78	2.28	**U = 170**	0.000*	14.10	1.18	11.78	2.28	U = 324.5	0.099
	No	14.79	3.63	14.23	3.56			14.83	2.64	14.23	3.56		
Do you have any chronic physical diseases?	Yes	-	-	-	-	-	-	-	-	-	-	**-**	**-**
	No	13.39	3.61	13.52	3.39			14.39	1.96	14.77	2.55		
“Is anyone from your family suffering from depression?”	Yes	15.00	0.00	14.67	1.86	U = 189.5	0.575	15.50	2.51	14.67	1.86	U = 185	0.495
	No	13.28	3.71	13.24	3.64			14.44	2.22	13.24	3.64		
Have you ever experienced thoughts of suicide?	Yes	-	-	-	-	-	-	-	-	-	-	-	-
	No	13.39	3.61	13.52	3.39			14.39	1.96	14.77	2.55		

K, Kruskal-Wallis test; U, Mann-Whitney test. *Significance level at P-value < 0.05; –No Statistic could be computed because one of the cells has a zero value

## Discussion

Our findings indicated that nature- based nursing intervention was successful in improving depressive symptoms, like insomnia, and feeling of hopelessness, both of which showed a reduction in severity. To the best of the information we have, this is the initial research to use non-pharmacological, nature-based nursing intervention for depressed patients complaining of insomnia and hopelessness. Adopting a nature-connected lifestyle, including regular exposure to natural environments, mindfulness in nature, and outdoor physical activities, can help to handle sleep disturbances and depressive symptoms, thereby improving overall quality of life.

**Concerning the socio-demographic data of patients in the studied groups**, the demographic characteristics of participants reflected a well-balance in the distribution across the nature-based intervention groups and the control groups. This balance provides a strong basis for making valid comparisons. All participants were males, with mean ages of 43.48 ± 7.47 years in the nature- based nursing intervention groups and 44.16 ± 8.58 years in the control group, and there was no statistically significant difference between the two. This indicates the demographics accessible during the period of data collection. This gave researchers a great chance to examine depression in a population that faces numerous obstacles in life and tends to repress emotional suffering.

Furthermore, as men often respond better to practical, engaging activities than to conventional verbal intervention, integrating nature-based nursing intervention with this group offered a greater knowledge of the efficacy of this intervention. In addition, the groups were also similar in terms of marital status, occupation, residence, and living arrangements, which suggests that these factors did not skew the study’s results. Minimal variations were seen in place of residence and educational level; however, they were statistically negligible (*p* > 0.05). The overall notice balance in these demographics appends confidence in attributing the apparent reduction in feeling of hopelessness and improvements in insomnia to nature-based nursing intervention.

**Concerning the medical history of depressed patients in the studied group**, the largest percentage of patients in both groups were hospitalized greater than once. This could be because of the chronic and recurring nature of depression as well as inadequate adherence to drugs, as it is often associated with severe side effects particularly affecting sexual function. In our study, where the sample consisted totally of males who, according to their own reports, were using selective serotonin reuptake inhibitors (SSRIs), this issue is especially significant, as males are more likely to cease drugs due to these side effects (sexual dysfunction), increasing their risk of relapse and repeated hospitalizations. This emphasizes the requirement for comprehensive intervention. Thus, nature-based nursing intervention may propose a more acceptable and sustainable complementary intervention for depressed patients who are hospitalized multiple times. where it is considered as an additional intervention that may augment the pharmacological effect. This result was in harmony with Catalini, et al. [40] who declared that males diagnosed with depression are vulnerable to recurrent hospitalization.

**Concerning insomnia in the studied groups**, the results of the current study displayed that, at post intervention and 2 months follow-up, a highly statistically significant variance was detected between nature-based groups and control groups for insomnia. This may be owing to activities of nature- based nursing intervention such as walking and jumping rope in the park, squat jumps, light jogging, and growing aromatic plants (jasmine and basil plants). These activities play a crucial role in regulating their natural sleep by releasing excess energy and enhancing the effects of melatonin, a hormone that regulates the body’s biological clock. In addition, these activities were implemented in the morning when the sun was shining, which in turn leads to better melatonin regulation and supports a healthy circadian rhythm. Consequently, this contributed to a noticeable reduction in insomnia levels, as measured by the insomnia subscale of the sleep disturbance scale. This finding was congruent with Zapalac et al. [41] who reported that light-to-moderate physical activity (walking, running, movement exercise) in naturalistic environments improved sleep architecture, including increased deep sleep and better sleep onset, which are linked to reductions in insomnia symptoms.

Likewise, the research of Kruk, Aboul-Enein, & Duchnik [42],; Kim, Ka & Park [43], & Lee, et al. [44]., also stated that participating in exercise enhances sleep quality through numerous processes. It boosts melatonin production, which aids sleep cycle control. It also diminished stress, which is a common obstruction for regular sleep. Furthermore, it aids sleep initiation through modulation of temperature. Thus, clinical and lifestyle recommendations suggest that practicing regular physical exercise in the morning, especially under natural light exposure, is useful for lowering insomnia and promoting healthy sleep patterns. Furthermore, a quasi- experimental study conducted by Shen, Hung, & Fang [45] demonstrated that participants who engaged in therapeutic horticulture reported improvements in subjective sleep quality. Also, Yang, et al., [46] discovered that plant material and horticultural activities were effective in improving the sleep function of studied subjects, especially sleep disturbances.

**Regarding hopelessness in the studied groups**, the outcomes of the current study reflected that, at post and 2 months follow-up, a highly statistically significant diversity was detected between nature- based and control groups for feeling hopelessness. The enhancement that was made may be attributed to the effect of nature-based nursing intervention, which included activities that fostered a sense of hope among participants through various mechanisms. For example, agricultural activities (sensory interaction with plants) and playing in nature helped revive a sense of life meaning and accomplishment, directly contributing to the enhancement of positive emotional experiences associated with hope. Furthermore, the use of sessions containing mindfulness, the “One Box” exercise, and the “Three Good Things” exercise helped patients change their thinking and interpretation of negative situations, transforming their suffering from weakness and despair into an opportunity for learning and a more optimistic outlook.

The improvement of post intervention outcomes noted in this study was in line with outcomes of Atta, Salama, & Menessy [47] who revealed that horticultural therapy program enhanced hope among patients with psychotic disorders. These outcomes were matched with Johnson et al. [48] who found that participants who spend more hour’s gardening or participating in outside recreational activities had a greater level of hope or a low level of hopelessness. Furthermore, the current study outcome corresponded with those Chu, Chan, & Chen [49] They concluded that participants who performed horticultural activity had mean score for “sense of hope” that elevated from3.28 beforehand the intervention to 3.81 points following it. They also reported that participants were floored by germination process and felt a sense of achievement from caring for the seedling, which further strengthened their hope.

**As regards correlations between feeling of hopelessness and insomnia**, the result of current research showed that feeling of hopelessness at post-intervention was positively correlated with insomnia post intervention and at two months follow-up. This means that improvements in insomnia were associated with reduced feeling of hopelessness over time. This could be due to the hormones responsible for regulating mood and emotions becoming unstable as a result of insomnia. Consequently, the patients’ experience loses their ability to think clearly when facing life challenges as well as difficulty regulating their emotions. Overtime, they focused more on pessimistic negative thoughts about themselves and their future, which worsened the sense of hopelessness. On the other hand, the more hopelessness they feel, the more they tend to overthink and be anxious at night, making it harder to fall asleep and trapping them in a sorrowful cycle of insomnia and emotional distress.

This perspective was shared by Benkirane et al. [50] who stated that increased sleep fragmentation was associated with worse executive function performance, irrespective of sleep duration. Similarly, a study done by Boon et al. [51] showed that sleep disturbances affect emotional regulation; meaning that people with interrupted sleep use ineffective ways to regulate their emotions, such as rumination, which leads to an increase in negative feelings. The significant correlation seen in the current study was consistent with a longitudinal study done by Kivelä, Van der Does, & Antypa [52]. They reflected that hopelessness mediates the relation between poor sleep (insomnia) and suicidal ideation, indicating that insomnia can intensify the sense of hopelessness. Furthermore, as demonstrated by Chang, Lai, & Fu [53] a higher level of insomnia severity was significantly associated with a higher level of hopelessness (coefficient = 0.0421, *p* < 0.001). These results highlight the magnitude of creating specific interventions aimed at enhancing sleep quality in patients diagnosed with depression, as such interventions can effectively contribute to alleviating feelings of hopelessness. Consequently, nature-based nursing interventions are considered promising for reducing feelings of hopelessness and improving sleep quality by promoting relaxation, reconstructing negative thoughts, and regulating emotional.

Additionally, a significant relation was noticed between insomnia and living arrangements (with family or alone) and marital status. This suggested that participants who live alone, or single are more vulnerable to sleep disturbance (insomnia). The interpretation of above finding may be due to feelings of loneliness and insecurity during nighttime. Otherwise, marital relationships or living with family can influence the occurrence of insomnia positively through feeling of security, a sense of belonging, and emotional support or negatively through domestic challenges and shared responsibilities. This finding was like the outcomes of Kim et al. [54]; they discovered being married was associated with fewer insomnia symptoms and better sleep health in general (F > 2.804, *p*< 0.044). In the same direction, this result was compatible with the results of Moon & You [55] who reported that adults living alone had a higher prevalence of insomnia compared to those living with others. Conversely, Othman, & Salem [56] revealed that there is no correlation found between marital status and insomnia. The opposition may be a return to the nature of the sample and the majority of participants of the previous study were married.

According to the outcomes of the current research, there was a substantial relation between insomnia and education level. This might be due to individuals with basic education being ordinarily employed in labor-rigorous work or skilled crafts that entail daily physical energy, and their work habitually features design schedules, helping in the tuning of their sleep-wake patterns. While individuals who have advanced education commonly face anxiety regarding job chances, pursuing graduation, and the horror of unemployment. This concern causes increased mental thinking at night and difficult relaxation, which is reflected in the form of insomnia. This result was incongruent with Bjørn Bjorvatn et al. [57] who illustrated that sleep health was better with higher educational levels. This discrepancy can be explained by differences in subjects and socioeconomic context. Higher education in European settings is different from Egyptian society, as it is associated with career stability and well-being.

Moreover, the present finding reflected that significant relations were seen between feeling of hopelessness and age. As participants aged 29–43 years showed a higher mean score contrasted with those aged 44years and above. This result reflected that the feeling of hopelessness tends to reduce slightly with age. This may be explained by individuals in middle age often complaining of greater pressure, which contributes to higher levels of stress and perceived hopelessness. On the other hand, older people have more life experience and emotional flexibility, which helps them to address life stressors which results in reducing senses of hopelessness. Despite most preceding experiments not directly exhibiting a relationship between age and hopelessness, this finding adds to scientific literature by emphasizing the potential role of age in influencing hopelessness levels.

The current finding also indicated that a significant association was observed between feeling of hopelessness and occupation. As working participants showed a higher mean score compared to those non-working. This could be because employed people have been exposed to low job satisfaction, occupational stress and financial challenges, which place them emotional exhaustion and pessimistic outlook. These results are compatible with Simard et al. [58]. who reported that a decreased sense of meaning in life is related to low job satisfaction, which in turn is associated with increased psychological distress and despair. Therefore, nature-based nursing interventions may help compensate for this deficiency by enhancing the sense of meaning, thus reducing levels of despair.

### Limitation of the study

One of the main constraints of this study is that only 62 individuals were studied, and all were male, a fact that might influence generalizing from the findings. In addition, the short duration of follow-up (two months) did not allow us to evaluate a valuation of the long-term sustainability of the impact. Another restraint is that the intervention was designed exclusively for patients with depression, preventing us from understanding whether benefits could extend to other mental health disorders. Further psychometric validation is also required for the structured interview questionnaire. On the same context, reduces the ability to establish full causal relationships as the study used a quasi-experimental design compared to randomized controlled trials. Moreover, the nature-based intervention was carried out during the summer season, which may have affected patients’ willingness to do outdoor nature-based activities. Lastly, the naturalistic activities used were only carried out in one health care facility in Egypt, which may constrain the applicability of the results to other settings or to other countries with different cultural backgrounds.

## Conclusion

The outcome findings of this research suggest that nature- based nursing intervention may have contributed to making patients with depression feel low hopelessness as well as improve in their insomnia across all levels (mild, moderate, and severe) as measured by the scale of sleep disorders. These constructive variations support the potential benefits of nature-based nursing interventions as a complement intervention to conventional care. However, the low sample size, using a single setting, and the quasi-experimental design, additional studies are necessary to verify the intervention’s efficacy more substantially.

### Research implication

The outcomes of this study have deep implications for nursing practice and health policy, especially when it pertains to mental health care services. Regarding nursing practice, psychiatric nurses are in a distinctive position to plan, organize, and carry out such innovative interventions and incorporate them into daily care plans due to their close contact with patients. Nature-based nursing interventions are cheap, non-pharmacological methods that can help with depression related problems, such as insomnia and feeling of hopelessness. Also, identical interventions in nearby urban green spaces or gardens can be conducted by nurses to enhance accessibility and continuity of care. Furthermore, they can help patients to adopt self-care practices after discharge.

Politically, incorporating nature-based nursing interventions into mental health care protocols is effective as a low-cost strategy that reduces the burden on health care systems. In addition, it is important that nature-based nursing interventions become part of public health plans by providing clear policies and practical legislation that support the creation and preservation of green spaces while ensuring that these spaces are available and accessible to everyone in the community.

Adding nature-based nursing interventions in psychiatric hospitals is a chance to provide comprehensive care and different treatment options for psychiatric patients. Furthermore, one of the biggest advantages of NBNI is that they tolerate patients’ needs. Furthermore, it encourages long-term recovery and boosts the efficacy of psychological services (e.g., support groups for depression patients who engage in group gardening activities).

### Recommendation and future research

Based on the research outcome, it is recommended that hospital health teams should integrate nature-based nursing activities in mental health care as a supportive intervention for patients with depression, alongside routine care to enhance holistic well-being and improve mental health outcomes. Nursing curricula should involve chapters on training on nature- based intervention to equip new graduate nurses with innovative practical skills for implementing such intervention in clinical practice. Furthermore, policymakers ought to promote the integration of this intervention into healthcare services via appropriate regulations and guidelines. Extra studies are required to investigate the effectiveness of different types of nature- based nursing intervention in other diagnoses, different seasons, both genders, using a randomized controlled design and across multiple settings. Furthermore, longer follow-up periods are essential to assess the sustainability of the observed improvement and to strengthen the potential for broader application.

## Supplementary Information

Below is the link to the electronic supplementary material.

Supplementary Material 1

## Data Availability

The datasets supporting the conclusion of this article are included within the article.

## Abbreviations

NBNI: Nature-based nursing intervention
BHS: Beck Hopelessness Scale

## References

[1] World Health Organization. Depression [Internet]. Geneva, World Health Organization. 2025 Jan 7 [cited 2025 Sep 6]. Available from: https://www.who.int/news-room/fact-sheets/detail/depression

[2] Ortega MA, Fraile-Martínez Ó, García-Montero C, Alvarez-Mon MA, Lahera G, Monserrat J, et al. Nutrition, epigenetics, and major depressive disorder: understanding the connection. Front Nutr. 2022;9:867150. 10.3389/fnut.2022.867150 PMC9158469 35662945

[3] Fang H, Tu S, Sheng J, Shao A. Depression in sleep disturbance: a review on a bidirectional relationship, mechanisms and treatment. J Cell Mol Med. 2019;23(4):2324–32. 10.1111/jcmm.14170. 30734486 10.1111/jcmm.14170 PMC6433686

[4] Plante DT. The evolving nexus of sleep and depression. Am J Psychiatry. 2021;178(10):896–902. 10.1176/appi.ajp.2021.21080821. 34592843 10.1176/appi.ajp.2021.21080821

[5] Banno M, Tsujimoto Y, Kohmura K, Dohi E, Taito S, Someko H, Kataoka Y. Unclear insomnia concept in randomized controlled trials and systematic reviews: a meta-epidemiological study. Int J Environ Res Public Health. 2022;19(19):12261. 10.3390/ijerph191912261. 36231555 10.3390/ijerph191912261 PMC9566752

[6] Marchetti I, Alloy LB, Koster EH. Breaking the vise of hopelessness: targeting its components, antecedents, and context. Int J Cogn Therapy. 2023;16(3):285–319. 10.1007/s41811-023-00165-1. 10.1007/s41811-023-00165-1 PMC11314313 39131585

[7] Hosseinzadeh Oskouei A, Sardarzehi R, Zamani Zarchi MS, Tavallaei Zavareh SA, Shams J, Kianimoghadam AS, Arani M, A. The mediating role of feelings of hopelessness and repetitive negative thinking in the relationship between perfectionism and depressive symptoms among medical students with suicidal ideation. Front Psychiatry. 2025;16:1560653. 10.3389/fpsyt.2025.1560653. 40433176 10.3389/fpsyt.2025.1560653 PMC12106920

[8] Wilkie S, Davinson N. The impact of nature-based interventions on public health: a review using pathways, mechanisms and behaviour change techniques from environmental social science and health behaviour change. J Br Acad. 2021;9:33–61. 10.5871/jba/009s7.03.

[9] Wilson EO. Biophilia: the human bond with other species. Cambridge (MA): Harvard University Press; 1984. ISBN: 9780674074422.

[10] Kaplan R, Kaplan S. The experience of nature: a psychological perspective. Cambridge University Press; 1989. ISBN: 9780521349394.

[11] Ulrich RS. Aesthetic and affective response to natural environment. Behavior and the natural environment. Boston, MA: Springer US; 1983. pp. 85–125. 10.1007/978-1-4613-3539-9_4.

[12] Howarth M, Brettle A, Hardman M, Maden M. What is the evidence for the impact of gardens and gardening on health and well-being: a scoping review and evidence-based logic model to guide healthcare strategy decision making on the use of gardening approaches as a social prescription. BMJ Open. 2020;10(7):e036923. 32690529 10.1136/bmjopen-2020-036923 PMC7371129

[13] Van van den Berg AE, Beute F. Walk it off! The effectiveness of walk and talk coaching in nature for individuals with burnout-and stress-related complaints. J Environ Psychol. 2021;76:101641. 10.1016/j.jenvp.2021.101641.

[14] Nguyen PY, Rahimi-Ardabili H, Feng X, Astell-Burt T. Nature prescriptions: a scoping review with a nested meta-analysis. medRxiv. 2022 Mar. https://www.medrxiv.org/content/. 10.1101/202203.23.22272674v1. Accessed 12 Apr 2022.

[15] Sia A, Tam WW, Fogel A, Kua EH, Khoo K, Ho RC. Nature-based activities improve the well-being of older adults. Sci Rep. 2020;10(1):18178. 10.1038/s41598-020-74828-w. 33097785 10.1038/s41598-020-74828-w PMC7585438

[16] Daut RA, Fonken LK. Circadian regulation of depression: A role for serotonin. Front Neuroendocrinol. 2019;54:100746. https://linkinghub.elsevier.com/etrieve/pii/S009130221930010X 10.1016/j.yfrne.2019.04.003 PMC9826732 31002895

[17] Harrison L, Coventry P, Armitt H, Chew-Graham C, Churchill R, Darcy P, White PC. Nature-based interventions for health and wellbeing: what works. York; 2023. Available from: https://www.researchgate.net/publication/375463122_Nature-based_interventions_for_health_and_wellbeing_what_works

[18] Keenan R, Lumber R, Richardson M, Sheffield D. Three good things in nature: a nature-based positive psychological intervention to improve mood and well-being for depression and anxiety. J Public Mental Health. 2021;20(4):243–50. 10.1108/JPMH-02-2021-0029.

[19] Berry C, Fountain J, Forbes L, Bogen-Johnston L, Thomson A, Zylko Y, Michelson D. Developing a hope-focused intervention to prevent mental health problems and improve social outcomes for young women who are not in education, employment, or training (NEET): A qualitative co-design study in deprived coastal communities in South-East England. PLoS ONE. 2024;19(5):e0304470. 10.1371/journal.pone.0304470. PMID: 38820387; PMCID: PMC11142577. 38820387 10.1371/journal.pone.0304470 PMC11142577

[20] Gruber R, Schwanda M. Hopelessness during acute hospitalization is a strong predictor of mortality. Evid Based Nurs. 2020. Retrieved December 17, 2021, from https://ebn.bmj.com/content/24/2/53. 10.1136/ebnurs-2019-103154 PMC8005791 32217643

[21] Tambyah R, Olcoń K, Allan J, Destry P, Astell-Burt T. Mental health clinicians’ perceptions of nature-based interventions within community mental health services: evidence from Australia. BMC Health Serv Res. 2022;22(1):841. 10.1186/s12913-022-08223-8. 35773704 10.1186/s12913-022-08223-8 PMC9244442

[22] Rabie AM, Sabry N, Noby S, Shaker MN, Ali M. National survey for mental health in Egypt: one-year prevalence of common mental disorders. Cairo: Research Unit, General Secretariat of Mental Health & Addiction Treatment, Ministry of Health and Population. 2017.

[23] Odejimi O, Tadros G, Sabry N. A systematic review of the prevalence of mental and neurocognitive disorders amongst older adults’ populace in Egypt. Middle East Curr Psychiatry. 2020;27(1):47. 10.1186/s43045-020-00055-8.

[24] Mo Y, Lei Z, Chen M, Deng H, Liang R, Yu M, Huang H. Effects of self-help mindfulness-based cognitive therapy on mindfulness, symptom change, and suicidal ideation in patients with depression: a randomized controlled study. Front Psychol. 2023;14:1287891. 10.3389/fpsyg.2023.1287891. 38106401 10.3389/fpsyg.2023.1287891 PMC10722434

[25] Calarco CA, Lobo MK. Depression and substance use disorders: clinical comorbidity and shared neurobiology. In international review of neurobiology. Acad Press. 2021;157:245–309. 10.1016/bs.irn.2021.03.008. 10.1016/bs.irn.2020.09.004 33648671

[26] Okasha T, Basu S, Hamed A, Bahieldin MM, Ramadan MA, Abdelwahab M, Mohamed O. PMH12 to evaluate the overall clinical and economic burden of treatment resistant depression in Egypt from the payers perspective. Value Health. 2021;24:S130. 10.1016/j.jval.2021.04.634.

[27] Brody DJ, Hughes JP. Depression prevalence in adolescents and adults: United States, August 2020-August 2023. US Department of Health & Human Services, Centers for Disease Control and Prevention, National Center for Health Statistics; 2025. 10.15620/cdc/174579.

[28] Buysse DJ, Reynolds III, Monk CF, Berman TH, S. R., Kupfer DJ. The Pittsburgh sleep quality index: a new instrument for psychiatric practice and research. Psychiatry Res. 1989;28(2):193–213. 2748771 10.1016/0165-1781(89)90047-4

[29] Rosner BA. Fundamentals of biostatistics. Volume 6. Belmont, CA: Thomson-Brooks/Cole; 2006.

[30] Zein-elabdeen AM, Ibrahim A, El-Bilsha M. Socio-demographic and clinical profile of patients with major depressive disorder in an Egyptian sample. Mansoura Nurs J. 2024;11(3).

[31] Dafallah AMM. The prevalence of depression in light of some demographic variables in sudan: an analytical study for the period (2000–2023). J Educational Sci Humanit. 2025;49565–92. 10.55074/hesj.vi49.1553.

[32] Beck AT, Weissman A, Lester D, Trexler L. The measurement of pessimism: the hopelessness scale. J Consult Clin Psychol. 1974;42(6):861. 4436473 10.1037/h0037562

[33] Michael A. The Beck hopelessness scale: standardization on Syrian samples. J Social Stud Univ Sci Technol. 2007;12(23):84–122. http://search.mandumah.com/Record/28745.

[34] Abdel-Halim AW. Sleep disorders and their relationship to psychological boredom and psychosomatic disorders among a sample of university youth. J Psychol Couns Ain Shams Univ Egypt. 2015;4451–101. 10.21608/CPC.2014.48994.

[35] Šorytė D, Rosa CD, Collado S, Pakalniškienė V. The effects of nature-based interventions on individuals’ environmental behaviors: protocol for a systematic review of controlled trials. Front Psychol. 2023;14:1145720. 10.3389/fpsyg.2023.1145720. 37333586 10.3389/fpsyg.2023.1145720 PMC10275608

[36] Rosa CD, Chaves TS, Collado S, Larson LR, Lee KJ, Profice CC. The potential of gardening and other plant-related interventions to reduce symptoms of depression: a systematic review of non-randomized controlled trials and uncontrolled studies. People Nat. 2025;7(1):295–316. 10.1002/pan3.10764

[37] Spahn L, Rosenblum L, Penzel T, Lederer K, Salanitro M, Fietze I. The influence of scent on sleep quality. J Biomed Res Environ Sci. 2022;3(10):1146–51. 10.37871/jbres1569. Article ID: jbres1569.

[38] Miraj S, Kiani S. Study of Pharmacological effect of ocimum basilicum: A review. Der Pharmacia Lettre. 2016;8:276–80.

[39] Karimi FZ, Hosseini H, Mazlom SR, Rakhshandeh H, Asadpour H. The effect of oral capsule of ocimum Basilicum leaf extract on sleep quality and insomnia severity in menopausal women: a randomized clinical trial. Phytother Res. 2023;37(6):2344–52. 10.1002/ptr.7753. 36750371 10.1002/ptr.7753

[40] Catalini A, Mazza C, Cosma C, Stacchini L, Caminiti M, Minutolo G, et al. The role of gender in the association between mental health and avoidable hospitalization. Eur J Public Health. 2022;32(Suppl 3):ckac131-496. 10.3390/ijerph192214691 PMC9690620 36429414

[41] Zapalac K, Miller M, Champagne FA, Schnyer DM, Baird B. The effects of physical activity on sleep architecture and mood in naturalistic environments. Sci Rep. 2024;14(1):5637. 10.1038/s41598-024-56332-7. 38454070 10.1038/s41598-024-56332-7 PMC10920876

[42] Kruk J, Aboul-Enein BH, Duchnik E. Exercise-induced oxidative stress and melatonin supplementation: current evidence. J Physiological Sci. 2021;71(1):27. 10.1186/s12576-021-00812-2. 10.1186/s12576-021-00812-2 PMC8409271 34470608

[43] Kim N, Ka S, Park J. Effects of exercise timing and intensity on physiological circadian rhythm and sleep quality: a systematic review. Phys Activity Nutr. 2023;27(3):52. 10.20463/pan.2023.0029. 10.20463/pan.2023.0029 PMC10636512 37946447

[44] Lee K, Hong KS, Park J, Park W. Readjustment of circadian clocks by exercise intervention is a potential therapeutic target for sleep disorders: a narrative review. Phys Activity Nutr. 2024;28(2):35. 10.20463/pan.2024.0014. 10.20463/pan.2024.0014 PMC11298283 39097996

[45] Shen JL, Hung BL, Fang SH. Horticulture therapy affected the mental status, sleep quality, and salivary markers of mucosal immunity in an elderly population. Sci Rep. 2022;12(1):10246. 10.1038/s41598-022-14534-x. 35715581 10.1038/s41598-022-14534-x PMC9205955

[46] Yang J, Deng Z, Pei S, Zhang N. A feasibility study on indoor therapeutic horticulture to alleviate sleep and anxiety problems: the impact of plants and activity choice on its therapeutic effect. Complement Ther Med. 2024;81:103032. 10.1016/j.ctim.2024.103032. 38452859 10.1016/j.ctim.2024.103032

[47] Atta MHR, Salama EA, Menessy RFM. Effect of horticultural therapy program on psychological Wellbeing, hope and social adjustment among patients with psychotic disorders: A nursing perspective. J Psychiatr Ment Health Nurs. 2025. 10.1111/jpm.13180 40515548

[48] Johnson M, Waliczek TM, Etheredge C, Bradley JC. The connection between gardening and outdoor activity during the COVID-19 pandemic and perceptions of hope, hopelessness, and levels of stress, anxiety, and depression. HortTechnology. 2023;33(2):168–75. 10.21273/HORTTECH05109-22.

[49] Chu HY, Chan HS, Chen MF. Effects of horticultural activities on attitudes toward aging, sense of hope and hand–eye coordination in older adults in residential care facilities. Int J Environ Res Public Health. 2021;18(12):6555. 10.3390/ijerph18126555. 34207071 10.3390/ijerph18126555 PMC8296344

[50] Benkirane O, Delwiche B, Mairesse O, Peigneux P. Impact of sleep fragmentation on cognition and fatigue. Int J Environ Res Public Health. 2022;19(23):15485. 10.3390/ijerph192315485. 36497559 10.3390/ijerph192315485 PMC9740245

[51] Boon ME, van Hooff MLM, Vink JM, Geurts SAE. The effect of fragmented sleep on emotion regulation ability and usage. Cogn Emot. 2023;37(6):1132–43. 10.1080/02699931.2023.2224957. 37337975 10.1080/02699931.2023.2224957

[52] Kivelä LM, Van der Does W, Antypa N. Sleep, hopelessness, and suicidal ideation: an ecological momentary assessment and actigraphy study. J Psychiatr Res. 2024;177:46–52. 10.1016/j.jpsychires.2024.06.039. 38972264 10.1016/j.jpsychires.2024.06.039

[53] Chang Q, Lai D, Fu Y. Mechanisms connecting insomnia to hopelessness among Chinese older adults: serial mediating roles of fatigue and social support. Int J Geriatr Psychiatry. 2022;37(6). 10.1002/gps.5720. 10.1002/gps.5720 35521653

[54] Kim Y, Ramos AR, Carver CS, Ting A, Hahn K, Mossavar-Rahmani Y, et al. Marital status and gender associated with sleep health among Hispanics/Latinos in the US: results from HCHS/SOL and Sueño ancillary studies. Behav Sleep Med. 2022;20(5):531–542. 10.1080/15402002.2021.1953499 10.1080/15402002.2021.1953499 PMC8784567 34308745

[55] Moon S, You SY. Prevalence and risk factors of insomnia among older adults living alone: A cross-sectional study based on spielman’s 3P behavioral model. Geriatr Nurs. 2025;66:103608. 10.1016/j.gerinurse.2025.103608. 40946404 10.1016/j.gerinurse.2025.103608

[56] Othman MN, Salem HA. Association between sleep disorders and psychiatric illnesses: A Cross-Sectional study. Aswan Univ Med J. 2025;5(1):92–101. 10.21608/aumj.2024.346315.1174.

[57] Bjorvatn B, Waage S, Pallesen S, Buysse DJ, Saxvig IW. The association between different sleep health dimensions and sex, age, education, circadian preference, and chronic insomnia: a representative population-based study. Sleep Adv. 2023;4(1):zpad041. 10.1093/sleepadvances/zpad041. 37954092 10.1093/sleepadvances/zpad041 PMC10635412

[58] Simard AA, Seidler ZE, Oliffe JL, Rice SM, Kealy D, Walther A, Ogrodniczuk JS. Job satisfaction and psychological distress among help-seeking men: does meaning in life play a role? Behav Sci. 2022;12(3):58. 10.3390/bs12030058. 35323377 10.3390/bs12030058 PMC8945795

