# Supplementary material for: Nature- based nursing intervention: the impact on insomnia and feeling of hopelessness among patients with depression at Nour El-Hikma psychiatric hospital, Egypt (A controlled quasi- experimental study)
Source: BMC Psychiatry. 2026 Feb 21;26:214. doi: 10.1186/s12888-026-07860-1 (PMC12934053; doi:10.1186/s12888-026-07860-1)
Supplement: Supplementary file 1 — Supplementary Material 1 [file 12888_2026_7860_MOESM1_ESM.docx]

**“Structured Interviewing Questionnaire for Demographic and Clinical Data – *Supplementary Material”***

**Name :**

**Code number:**

**Age:** ≤28.( ) 29 – 43 ( ) ≥44 ( )

**Sex :** Male ( ) Female ( )

**Education :**Basic education ( ) Secondary education ( ) University education ( )

**Marital status:**

- Single ( )
- Married ( )
- Divorced ( )
- Widowed ( )

**Occupational Status:**

- Employed ( )
- Unemployed ( )

**Residence:** Urban ( ) Rural ( )

**Living Situation**

- Do you live with your family? Yes ( ) No ( )
- Do you live alone? Yes ( ) No ( )

**Patient Admission History:**

- First admission to a psychiatric hospital ( )
- Repeated admission (More than one admission )to a psychiatric hospital ( )

**Medical History:**

- Do you have any chronic physical disability? Yes ( ) No ( )

**Family History:**

Is anyone from your family suffering from depression? Yes ( ) No ( )

**History of Suicidal Ideation**

**Have you ever experienced thoughts of suicide?**

- - Yes ( )
  - No ( )
